# Correlation between medical student empathy and a Korean nationwide comprehensive clinical assessment score at a medical school in Korea

**DOI:** 10.1097/MD.0000000000029497

**Published:** 2022-07-29

**Authors:** Min Kyu Jung, Sanghee Yeo, Won Kee Lee

**Affiliations:** a Department of Internal Medicine, School of Medicine, Kyungpook National University, Daegu, Republic of Korea; b Division of Gastroenterology and Hepatology, Department of Internal Medicine, Kyungpook National University Hospital; c Center for Medical Education, School of Medicine, Kyungpook National University, Daegu, Republic of Korea; d Department of Medical Informatics, School of Medicine, KyungPook National University, Daegu; e Medical Research Collaboration Center in Kyungpook National University Hospital.

**Keywords:** empathy, medical education, educational measurement, emotional intelligence

## Abstract

Empathy is the ability to understand and communicate a patient’s situation, perspective, and feelings. When demonstrated by healthcare professionals, this can improve patient adherence, satisfaction, and treatment outcomes. Empathic students have stronger affective skills and can acquire, develop, reinforce, and display strong affective behaviors, abilities, and attitudes.

We measured student empathy using the Student Version of the Jefferson Scale of Empathy (JSE-S) and assessed 3-year sequential clinical comprehensive assessment scores conducted by the Korean Medical Education Assessment Corporation to determine the relationship between JSE-S and clinical comprehensive assessment scores.

The study population comprised of 80 males (74%) and 28 females (26%), among which 38 (35%) and 62 (57%) students wanted to become private physicians and attending faculty, respectively. Regarding future majors, 58 students (54%) considered medical fields, whereas 40 students (37%) considered surgical fields. No significant differences in Korean JSE-S were observed according to medical student gender, career aspirations, or future major fields.

The modified Korean version of the JSE-S has 18 items. Item-total score correlations and Cronbach α evaluated the internal consistency reliability of the scale. The reliability of the Korean JSE-S was 0.910 by Cronbach α coefficient.

Female students had better scores than males. Students who wanted to be an attending faculty had better scores than others who wanted to be private physicians; however, these findings were not statistically significant. Significantly higher scores were seen among students aspired to work in the medical field (65.6 ± 8.8) versus in the surgical field (60.4 ± 8.2) in their 5th year (*P* < .01).

We were unable to show the positive correlations between the empathy scale and comprehensive assessment results. Among female medical students, comprehensive assessment results were inversely correlated with empathy toward the patient, but this was not statistically significant.

The modified Korean JSE-S has acceptable reliability. Every student had a better comprehensive assessment after studying the medical curriculum between the 4th and 6th years. The current nationwide assessment tool does not measure student empathy.

## 1. Introduction

Empathy is the ability to understand and communicate a patient’s situation, perspective, and feelings.^[1]^ Empathy plays an important role in a positive physician–patient relationship, wherein patient satisfaction can contribute to optimal clinical outcomes.^[2,3]^ Because of this, medical education has always valued empathy as an essential professional attribute of physicians.^[4]^ However, recent studies have found that empathy scores decline in medical school.^[5]^ Unfortunately, current medical education focuses more on detachment and objectivity rather than on the physician-patient relationship.^[6]^

Empathy for the patient could motivate students to study hard in order to take care of their own patients, as well as their clinical curiosity to solve the patients’ problems. It can be a very strong motive to learn about clinical diseases.

The Student Version of the Jefferson Scale of Empathy (JSE-S) was specifically developed as a self-reported scale for assessing empathy in medical students.^[7]^ The JSE-S includes 20 items that measure 3 underlying empathy constructs (i.e., perspective taking, compassionate care, and standing in patient’s shoes) that have been proven to have good psychometric properties.^[7–9]^ In South Korea, the Korean JSE-S is used to measure empathy toward patients.^[10–12]^ After a statistical item analysis, 2 items were not deemed useful because of Korean cultural influences.^[12]^ This study used the 18 items from the original Korean JSE-S.^[12]^

Korea has 40 medical schools with approximately 3000 students. Each medical school voluntarily participates in the Korea Association of Medical Colleges (KAMC). The KAMC supports the Medical Education Assessment Corporation (MEAC) as they handle the clinical comprehensive assessment twice a year since 2009. In fact, 40 medical schools have used these assessments to identifiy students with learning difficulties.

We tried to evaluate the correlation between students’ empathy and clinical compentence to solve the patients’ problem. This study aimed to determine the relationship between Korean JSE-S and clinical comprehensive assessment scores from the MEAC.

## 2. Participants and Procedures

A total of 108 medical students in South Korea participated in this cross-sectional study conducted in May 2020. The study participants were 6th-year medical students enrolled in the 6-year medical course at Kyungpook National University School of Medicine (KNUSM). We also assessed future career aspirations. The response rate was 99%. The demographic data of all students and respondents are presented in Table 1. Clinical comprehensive assessments were performed 4 times: in November 2018, November 2019, August 2020, and December 2020. This study was approved by the Institutional Review Boards of Kyungpook National University. All methods were carried out in accordance with relevant institutional and national guidelines and regulations for human research standards. Informed consent was obtained from all participants, who were over 16 years of age.

**Table 1:** Basic characteristics of the participants.

Characteristic		Frequency (%)	JSE-S score*
Sex	Man	80 (74.1)	92.7 ± 14.4
	Woman	28 (25.9)	91.2 ± 12.0
Career aspirations	Private physician	38 (35.2)	93.7 ± 13.5
	Attending faculty	62 (57.4)	91.7 ± 13.7
	Others	8 (7.4)	89.8 ± 16.3
Future major	Medical	58 (53.7)	90.0 ± 13.2
	Surgical	40 (37.0)	95.3 ± 14.0
	Others	10 (9.3)	93.6 ± 14.8

The medical curriculum at KNUSM is comprised of 3 years of preclinical work followed by 3 years of clinical work. Major clinical rotations are scheduled during the 5th year, while minor clinical rotations happen during the 6th year. The curriculum at KNUSM is typical of most medical schools in South Korea and similar to those in the United States and Canada. However, unlike the United States and Canada where medical schools are graduate schools, most Korean medical schools are undergraduate schools. A 4-year medical curriculum is preceded by a 2-year premedical course, totaling 6 years of medical training. Therefore, Korean medical students are likely to be younger than US/Canadian medical students.

### 2.1. Study Questionnaire

The Korean JSE-S was used to measure physician empathy.^[10–12]^ A standard back-translation procedure was performed. The JSE-S was translated into Korean, then back-translated into English. Korean and English language specialists reevaluated the accuracy of the translation. The Korean-translated version was a self-reported questionnaire consisting of 18 items with a 7-point Likert scale ranging from 1 (strongly disagree) to 7 (strongly agree), including 8 reverse items scored from 1 (strongly agree) to 7 (strongly disagree). The original version has 20 items. However, 2 (items 18 and 19) were excluded in this measurement tool as they were considered inappropriate for cultural reasons, resulting in a final total of 18 items.^[10–12]^ The modified Korean version of the JSE-S has 18 items with the following 3 components: “perspective taking” (items 2, 4, 5, 9, 10, 13, 15, 16, 17, and 20); “compassionate care” (items 1, 7, 8, 11, 12, and 14); “standing in patient’s shoes” (items 3 and 6).^[12]^

The total score was obtained by summing all items (maximum score = 126), where higher values indicated a higher degree of empathy.

We evaluated 3-year sequential clinical comprehensive assessment scores from written tests, with similar results to the Korean Medical Licensing Examination. The KAMC has been united to guide all medical school to build up the minimal requirements of medical education. It supports the MEAC when they carry out the comprehensive assessment, which consisted of 109 physician encounter situations. These include approaches to jaundice, chest pain, abdominal discomfort, headache, vaccination, weight loss, diarrhea, constipation, menorrhagia, and other clinical presentations. The clinical comprehensive assessment has been conducted twice a year since 2009. In 2019, a total of 10,357 students across 40 medical schools were tested. In KNUSM specifically, total of 108 medical students have taken the examination in their 4th, 5th, early- 6th, and late- 6th years of medical school.

### 2.2. Statistical Analyses

The mean JSE-S scores were compared using ANOVA or independent t-test. In cases of significance following ANOVA, post hoc comparisons were analyzed using the Tukey–Kramer method. Correlations between empathy and comprehensive assessment results were calculated via Pearson correlation coefficient. Tool reliability was evaluated via Cronbach α. SAS 9.4 (SAS Institute Inc., Cary, NC, USA) was used for the statistical analyses. P-values <0.05 were considered statistically significant.

## 3. Results

This study population comprised of 80 males (74%) and 28 females (26%). Among them 38 students (35%) wanted to become private physicians, while 62 (57%) wanted to become attending faculty. Regarding future majors, 58 students (54%) were interested in medical fields, whereas 40 students (37%) were interested in surgical fields. However, no significant differences in Korean JSE-S scores were observed between those with medical student gender, career aspirations, or future major fields (Table 1).

The modified Korean version of the JSE-S has 18 items with the following 3 components: “perspective taking”, “compassionate care”, “standing in patient’s shoes”.^[12]^ The 3-component Korean JSE-S is not different according to the basic characteristics (Table 2).

**Table 2:** Each component’s scores of the Korean JSE-S according to the basic characteristics

		Standing in patient’s shoes			Compassionate care			Perspective taking			Total		
Characteristic		Mean ± SD	t/F	p	Mean ± SD	t/F	p	Mean ± SD	t/F	p	Mean ± SD	t/F	p
Gender	Man	8.4 ± 2.3	-0.92	0.362	30.1 ± 6.3	1.08	0.283	54.3 ± 8.5	0.25	0.8	92.7 ± 14.4	0.49	0.627
	Woman	8.8 ± 2.4			28.6 ± 6.1			53.8 ± 8.1			91.2 ± 12.0		
Career aspirations	Private physician	8.7 ± 2.4	0.98	0.379	30.3 ± 6.0	0.33	0.722	54.7 ± 8.8	0.4	0.674	93.7 ± 13.5	0.37	0.692
	Attending faculty	8.2 ± 2.3			29.4 ± 6.5			54.1 ± 7.9			91.7 ± 13.7		
	Others	9.3 ± 2.3			28.8 ± 5.2			51.8 ± 10.1			89.8 ± 16.3		
Future major	Medical	8.3 ± 2.2	0.44	0.648	28.7 ± 6.1	2.02	0.138	53.0 ± 8.2	1.26	0.289	90.0 ± 13.2	1.82	0.167
	Surgical	8.7 ± 2.5			31.2 ± 5.4			55.3 ± 8.2			95.3 ± 14.0		
	Others	8.7 ± 2.8			28.8 ± 9.2			56.1 ± 9.9			93.6 ± 14.8		

Item-total score correlations and Cronbach α were used to evaluate the internal consistency reliability of the scale. The reliability of the Korean JSE-S was 0.910 by Cronbach α coefficient (Table 3), and the level of reliability was acceptable.

**Table 3:** Item-total score correlation of the Korean JSE-S

Cronbach coefficient alpha			Raw	0.901
			Standardized	0.91
Cronbach coefficient alpha with deleted variable				
	Raw variables		Standardized variables	
Deleted variable	Correlation with total	Alpha	Correlation with total	Alpha
Item 1	0.322	0.908	0.312	0.913
Item 2	0.628	0.894	0.653	0.903
Item 3	0.547	0.896	0.53	0.907
Item 4	0.652	0.893	0.673	0.903
Item 5	0.405	0.9	0.427	0.909
Item 6	0.303	0.903	0.276	0.914
Item 7	0.627	0.893	0.602	0.905
Item 8	0.66	0.892	0.639	0.904
Item 9	0.682	0.892	0.704	0.902
Item 10	0.591	0.894	0.619	0.904
Item 11	0.692	0.891	0.668	0.903
Item 12	0.643	0.892	0.621	0.904
Item 13	0.807	0.889	0.829	0.898
Item 14	0.205	0.906	0.192	0.916
Item 15	0.479	0.898	0.497	0.908
Item 16	0.742	0.891	0.769	0.9
Item 17	0.667	0.892	0.689	0.902
Item 18	0.6	0.894	0.629	0.904

In general, female students had better scores than males (Table 4). Students who wanted to be an attending faculty also had better scores than others who wanted to be a private physician (Table 4). However, these findings were not statistical significant. On the other hand, students who aspired to work in the medical field (65.6 ± 8.8) had significantly higher scores than those who aspired to work in the surgical field (60.4 ± 8.2) when they were in their 5th year (*P* < .01). This trend was also seen in the 4th year (48.0 ± 9.0 vs 44.9 ± 8.2), early 6th year (79.1 ± 8.1 vs 75.5 ± 8.6), and late 6th year (78.3 ± 8.5 vs 74.8 ± 7.7), but these findings were not statistical significant. Students generally had better comprehensive assessment scores in their 6th year versus in their 4th year (Table 4). Students interested in medical fields achieved better results than those interested in surgical or other fields.

**Table 4:** Korean nationwide clinical comprehensive assessment results according to basic characteristics.

		4th year			5th year			Early 6th year			Late 6th year		
		Mean ± SD	t/F	*P*	Mean ± SD	t/F	*P*	Mean ± SD	t/F	*P*	Mean ± SD	t/F	*P*
Gender	Man	45.9 ± 9.3	-1.69	0.096	62.7 ± 9.5	-0.73	0.467	77.0 ± 8.8	-0.94	0.35	76.0 ± 8.2	-1.06	0.291
	Woman	48.6 ± 6.5			64.2 ± 8.4			78.9 ± 9.4			78.0 ± 9.3		
Career aspirations	Private physician	45.0 ± 7.1	1.68	0.191	62.3 ± 9.2	0.5	0.609	77.5 ± 9.0	1.93	0.15	76.2 ± 8.2	1.83	0.166
	Attending faculty	47.9 ± 8.7			63.8 ± 8.6			78.3 ± 8.1			77.4 ± 7.8		
	Others	44.3 ± 14.0			61.4 ± 13.4			71.7 ± 13.9			71.4 ± 13.9		
Future major	Medical	48.0 ± 9.0	1.58	0.21	65.6 ± 8.8	4.83	**0.01**	79.1 ± 9.1	2.01	0.14	78.3 ± 8.5	2.71	0.071
	Surgical	44.9 ± 8.2			60.4 ± 8.2			75.5 ± 8.6			74.8 ± 7.7		
	Others	45.7 ± 8.4			59.7 ± 11.8			76.5 ± 8.5			73.7 ± 10.0		

We were unable to show the positive correlations between the empathy scale and comprehensive assessment results. Among female medical students, comprehensive assessment results were inversely correlated with empathy toward the patient, but this was not statistically significant (Table 5).

**Table 5:** Correlation between the empathy and comprehensive assessment results according to basic characteristics.

Basic characteristics		School year	Standing in patient’s shoes	Compassionate care	Perspective taking	Total
Total		4th	0.025	0.111	0.048	0.084
		5th	0.068	0.145	-0.101	0.016
		Early 6th	-0.013	0.024	-0.093	-0.048
		Late 6th	-0.014	0.029	-0.109	-0.055
Gender	Man	4th	-0.06	0.095	0.09	0.085
		5th	-0.111	0.057	-0.064	-0.031
		Early 6th	-0.09	0.036	0.022	0.014
		Late 6th	-0.123	0.03	0.016	0.003
	Woman	4th	0.309	0.269	-0.115	0.122
		5th	0.626†	0.474*	-0.221	0.22
		Early 6th	0.17	0.018	-0.418*	-0.238
		Late 6th	0.234	0.057	-0.444*	-0.223
Career aspirations	Private physician	4th	0.366*	0.249	0.013	0.184
		5th	0.242	0.302	-0.195	0.049
		Early 6th	0.183	0.066	-0.153	-0.039
		Late 6th	0.242	0.189	-0.069	0.081
	Attending faculty	4th	0.006	0.125	0.164	0.155
		5th	0.049	0.15	0.02	0.092
		Early 6th	0.005	0.084	0.107	0.103
		Late 6th	-0.024	0.043	0.024	0.03
	Others	4th	-0.585	-0.276	-0.363	-0.394
		5th	-0.324	-0.468	-0.364	-0.419
		Early 6th	-0.599	-0.572	-0.836†	-0.783†
		Late 6th	-0.582	-0.65	-0.837†	-0.806*
Future major	Medical	4th	0.095	0.201	0.138	0.195
		5th	0.152	0.254	0.06	0.18
		Early 6th	0.023	0.003	-0.135	-0.079
		Late 6th	0.006	0.042	-0.124	-0.057
	Surgical	4th	-0.193	-0.039	0.013	-0.042
		5th	-0.153	0.049	-0.054	-0.04
		Early 6th	-0.094	0.18	0.108	0.115
		Late 6th	-0.077	0.14	0.082	0.087
	Others	4th	0.654*	0.367	-0.064	0.309
		5th	0.596	0.31	-0.544	-0.06
		Early 6th	0.298	0.015	-0.317	-0.148
		Late 6th	0.260	-0.010	-0.311	-0.166

## 4. Discussion

This study utilized item-total score correlations and Cronbach α to evaluate the internal consistency reliability of the Korean JSE-S. This had a reliability of 0.910 by Cronbach α coefficient despite including only 18 items. All students had better comprehensive assessment scores in their 6th year compared to in their 4th year. Students interested in medical fields achieved better results than those interested in surgical or other fields. Among female medical students, comprehensive assessment results were inversely correlated with empathy toward the patient. However, we were unable to determine positive correlations between the empathy scale and comprehensive assessment results.

The Korean JSE-S appeared to have good quality and satisfactory factorial validity.^[10–13]^ Factor analyses affirmed that the Korean JSE-S had a 3-component figure structure comparative to that of the initial English version.^[9]^ Two items (items 18 and 19) were excluded from the original 20 items in the measurement tool used in this study since these were considered inappropriate for cultural reasons.^[12]^ However, the modified Korean JSE-S also had positive and statistically significant item-total score correlations, demonstrating that the direction of scoring was correct for all items, and each item significantly contributed to the total score. These results affirm that the Korean JSE-S is a valuable tool for surveying empathy in Korean medical students and can be used to recognize significant components for effective empathy education in future studies. A previous study reported a Cronbach α coefficient of 0.84,^[10]^ but the present study had a Cronbach α coefficient of 0.910, demonstrating its reliability despite including only 18 items.

The previous empathy scores of KNUSM students in 2014 and 2015^[12]^ were remarkable different from those in 2020. The empathy scores were higher than previous (92 vs 78), there was no clear explanation how the students improved their empathy scores. However, KNUSM adopted (1) a clinical communication skill program, which improved students’ patient communication skills, as well as (2) a patient-physician relationship program, which improved their understanding and thinking as the patient’s view in the clinical skill center. Nonetheless, the JSE-S scores of Korean medical students were lower than those of American, Mexican, and Polish students after adjusting the total score.^[9,14,15]^ Cross-cultural differences in standards, ethnicity, devout convictions, and sex stereotyping can influence empathic engagement in clinical situations. Asians are suggested to have a more collectivistic and less individualistic social culture than Westerners.^[16]^ Furthermore, Asian physicians also tend to adopt a more paternalistic role within the physician–patient relationship. Such sociocultural characteristics that extend to general medical practice in Asia may contribute to patients being less self-assured and physicians becoming more dictatorial. Potentially, this can result in a less patient-centered and empathic approach by such physicians. Previous studies have proposed that individual medical college cultures can also influence medical students’ empathy,^[17]^ similar to the findings of our study.

Clinical comprehensive assessment scores conducted by the Korean MEAC at every Korean medical school are typically used to evaluate the ability of medical students to perform physician encounters. The test is held at most Korean medical institutes twice a year. Thus, it represents a well-established, nationwide assessment tool for medical students. However, in this study, the written test results did not reflect students’ empathy toward patients despite the ability of the test to differentiate talented from underachieving students.

KNUSM is a typical medical school in Korea that demands high student competence, fosters high levels of competitiveness among colleagues, and emphasizes research and charitable care. Such institutional characteristics may have been a contributing factor to the low empathy scores of students enrolled in this study. Indeed, empathy scale results were found to be inversely correlated with the assessment results among female medical students. This finding may be due to the excessive study load, a lack of time, and an educational environment that emphasizes objective and scientific-based thought and reasoning.^[1]^

Women have greater empathy than men, possibly as they esteem interpersonal relationships and have a more thoughtful understanding of emotions and caring attitude in many studies.^[7,18,19]^ Moreover, women outscored men in a US physician study of empathy, in spite of the fact that the difference observed was not statistically significant.^[9]^ On the other hand, there was no difference between female and male medical students and dentistry students in a Polish study.^[14,15]^ In contrast, the present study showed that female students had lower empathy scores than male students, although the difference was not statistically significant.

This study has some limitations. First, the survey was conducted at a single medical school in Korea, which possibly limits the generalization of our results to Korean medical students. However, the medical educational program in KNUSM is common among most medical schools in South Korea. The Korean students also have a single ethnicity and similar educational background. Thus, our results could be generalized to other Korean medical schools in this aspect. Second, we utilized only a self-rating of empathy. Despite the fact that the JSE is reported to be well-correlated with observer ratings, self-reported results may have been subjected to biases and inconsistencies between self-reflection and actual behavior^[2]^ Third, this study didn’t conduct longitudinally. However, it was follow up study of every student who sent the agreement of participation in this study. We reviewed the student’s clinical comprehensive assessment score when they were 4th year. We evaluated the student’s Korean JSE-S and their clinical comprehensive assessment score when they were 5th year. One year later we checked their clinical comprehensive assessment score when they were 6th year. Their clinical comprehensive assessment score with Korean JSE-S were compared. Future studies are required to explain the role of culture and the impact of the medical educational programs on empathy.

The findings of this study demonstrate that empathy scale scores do not correlate with clinical comprehensive assessment scores. All Korean medical institutes emphasize teaching emotional intelligence and skills, including communicating and sympathizing with one another, but there is currently a lack of tools for assessing empathy. In these circumstances, a student may neglect the importance of ethics and empathy toward a patient. With medicine becoming increasingly dependent on artificial intelligence, current and future medical students will need to achieve high levels of empathy and emotional intelligence, which cannot be fully comprehended or substituted by artificial intelligence. Accordingly, there is a need to develop assessment tools to evaluate empathy and emotional intelligence among medical students.

## Authors’ contributions

As the corresponding author Min Kyu Jung designed and conducted this study and wrote the entire manuscript. Sanghee Yeo contributed to the study design and assisted with manuscript preparation. Won Kee Lee prepared data tables and performed statistical analyses. All authors reviewed the manuscript.

## Footnotes

* Mean ± SD.

* *P* < .05.

† *P* < .01.
